# iProL: identifying DNA promoters from sequence information based on Longformer pre-trained model

**DOI:** 10.1186/s12859-024-05849-9

**Published:** 2024-06-25

**Authors:** Binchao Peng, Guicong Sun, Yongxian Fan

**Affiliations:** School of Computer Science and Information Security, Guilin University of Electronic Technology, Guilin, 541004 China

**Keywords:** Promoter, Longformer, Deep learning, Natural language processing

## Abstract

Promoters are essential elements of DNA sequence, usually located in the immediate region of the gene transcription start sites, and play a critical role in the regulation of gene transcription. Its importance in molecular biology and genetics has attracted the research interest of researchers, and it has become a consensus to seek a computational method to efficiently identify promoters. Still, existing methods suffer from imbalanced recognition capabilities for positive and negative samples, and their recognition effect can still be further improved. We conducted research on *E. coli* promoters and proposed a more advanced prediction model, iProL, based on the Longformer pre-trained model in the field of natural language processing. iProL does not rely on prior biological knowledge but simply uses promoter DNA sequences as plain text to identify promoters. It also combines one-dimensional convolutional neural networks and bidirectional long short-term memory to extract both local and global features. Experimental results show that iProL has a more balanced and superior performance than currently published methods. Additionally, we constructed a novel independent test set following the previous specification and compared iProL with three existing methods on this independent test set.

**Supplementary Information:**

The online version contains supplementary material available at 10.1186/s12859-024-05849-9.

## Introduction

Promoters are important components of the DNA sequence, ranging from tens to thousands of base pairs in length. They are usually located in the vicinity of the gene TSS [1]. The promoter has a crucial role in regulating the activation or repression of transcription of specific genes in biological cells by binding to RNA polymerase to ensure that DNA transcription proceeds normally. In bacteria, for instance, cells regulate their transcription programs by adjusting RNA polymerase activity and altering the combination of promoters to which RNA polymerase can bind, thereby adapting to changing environments [2]. In eukaryotes, promoters consist of three promoter regions: core promoter, proximal promoter, and distal promoter [3]. The core promoter is the smallest promoter region, and the TATA-box is one of its most prominent elements. In prokaryotes, functionally specific $\sigma$ factors have varying degrees of preference for different promoters, and $\sigma$ factors and RNA polymerases recognize specific promoters and bind specific regions of the promoter to initiate transcription [4, 5].

In the past decade, computational methods and wet experiments have commonly identified *E. coli* promoters. However, with the rapid development of high-throughput sequencing technology, access to vast amounts of gene sequences has exploded. The time-consuming and expensive experimental methods have been brutal to support the processing of biological sequence data of this magnitude. Therefore, the search for a fast and efficient computational method for accurately identifying promoters has become the current research focus among many researchers in bioinformatics. According to statistics, more than 50 promoter benchmark datasets have been published since 2000, and there are also hundreds of computational methods for prokaryotic or eukaryotic promoter identification [6]. Overall, these computational methods can be roughly classified into three categories according to the tasks: promoter identification, promoter type identification, and promoter strength identification.

Some researchers have developed prediction models to identify ${\sigma }^{70}$ promoters, such as IBPP [7] based on evolutionary patterns and iPro70-PseZNC [8] based on pseudo nucleotide compositions. There are also two methods based on combining multiple features, namely70ProPred [9] and Sigma70Pred [10]. Other researchers have started to propose prediction models for identifying promoters and their type or strength. For example, computational methods such as iPromoter-2L [11], MULTiPly [12], iPromoter-BnCNN [13], iPro2L-PSTKNC [14], pcPromoter-CNN [15] and Expositor [16], as well as the location-based feature PPred-PCKSM [17] and the multi-source feature fusion-based PredPromoter-MF(2L) [18], predicted promoter types. In addition, Xiao et al. presented a benchmark dataset and the first two-layer prediction model iPSW(2L)-PseKNC, for predicting promoters and promoter strengths in 2019 [19]. This two-layer prediction model first uses PseKNC for feature encoding and then uses support vector machine (SVM) for prediction. Subsequently, from 2019 to 2022, researchers successively proposed computational methods with better prediction based on the dataset constructed by Xiao et al.: CNN-FastText [20], iPSW(PseDNC_DL) [21], iPromoter-ET [22], dPromoter-XGBoost [23] and BERT-Promoter [24]. The model CNN-FastText classifies promoters via deep learning and a combination of continuous FastText N-grams. The model iPSW(PseDNC_DL) uses convolutional neural networks to automatically learn sequence features and combines pseudo dinucleotide composition (PseDNC) to identify promoters and their strength. The method iPromoter-ET Identify promoters and their strength by extremely randomized tree-based feature selection. The method dPromoter-XGBoost uses four feature extraction methods and analysis of variance ANOVA for feature selection, and finally XGBoost for recognition. The BERT-Promoter method encodes DNA sequences using the BERT pre-training model, uses SHAP for feature selection, and uses different machine learning methods to predict promoter and promoter strength.

In our study, we have studied the sequence recognition of *E. coli* promoters in detail. We attempted two pre-trained models based on the attention mechanism of the NLP field, BERT [25] and Longformer [26], as well as different CNN structures, to propose a deep learning framework based on Longformer pre-trained model, iProL. In iProL, DNA sequences are treated as natural sentences and tokenized as the input of the model. After Longformer embedding, the obtained promoter DNA vector is fed into CNN and BiLSTM. Finally, the feature vector is fed to the full connected layer, and the prediction result is output. Experimental results show that iProL, which does not rely on any biological features, outperforms the state-of-the-art methods in identifying promoter sequences of *E. coli* through five-fold cross-validation. The source codes and datasets for the promoter predictions have been uploaded to https://github.com/20032303092/iProL.

## Materials and methods

### Prediction framework of the proposed iProLs

**Fig. 1:**
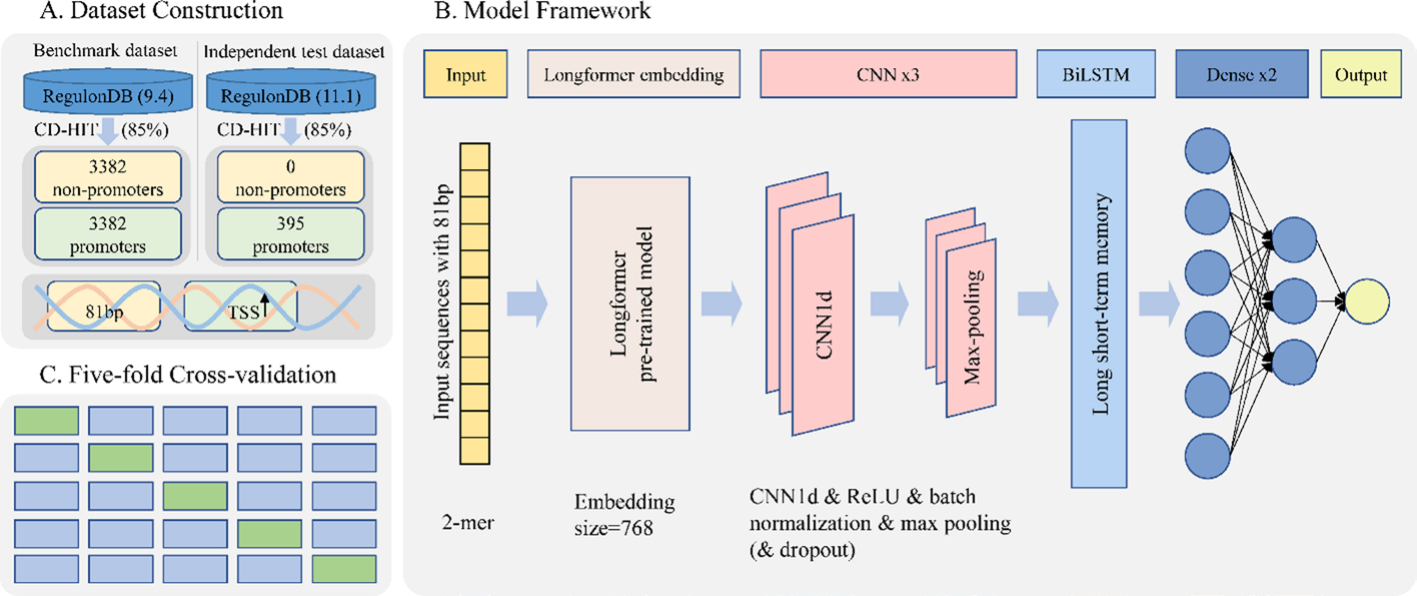
iProL overview. It includes **A** dataset construction, **B** model framework, and **C** five-fold cross-validation, where the data from the benchmark dataset and the independent test dataset do not overlap

In our study, we propose iProL, an advanced promoter prediction tool. Figure 1 depicts our entire experimental framework, including three parts: dataset construction, model structure, and five-fold cross-validation. The dataset construction part was fully described in the next section, and here we focus on the model architecture. As shown in part B of Fig. 1, iProL mainly consists of the input layer, Longformer embedding layer, CNN layer, BiLSTM layer, fully connected layer, and output layer. First, the input layer receives 81-bp long DNA sequences, split into 2-mer nucleotide segments with a stride of 1 before being fed into the model. Next, after processing through the Longformer embedding layer, we obtain DNA embedding vectors with dimensions of 79 × 768. Due to the large number of parameters in the Longformer pre-trained model and the limited size of our dataset, we avoided overfitting by not fine-tuning the pre-trained model. Subsequently, a three-layer one-dimensional CNN and BiLSTM form our feature extractor for obtaining 96-dimensional feature vectors. The three one-dimensional CNN layers have output channels of 128, 64, and 32, respectively, while the BiLSTM layer has a hidden size of 16 with one layer. Each one-dimensional convolution layer is followed by a batch normalization layer, ReLU activation function, max pooling layer, and dropout layer in sequence, except for the third convolution layer, which does not use a dropout layer. Finally, we feed the 96-dimensional feature vector into two fully connected layers activated by the ReLU function and the Sigmoid function, respectively, and then obtain the prediction result at the output layer. As shown in part C of Fig. 1, we evaluate the proposed model using five-fold cross-validation.

### Datasets

Our experimental data are derived from RegulonDB (version 9.4) [27]. This database is mainly used to store transcriptional regulatory sequences of the *E. coli* K-12 genome. Complete data can be downloaded from the official website of RegulonDB (available at https://regulondb.ccg.unam.mx). In this study, a benchmark dataset consistent with BERT-Promoter was used to facilitate comparison with the latest computational methods [23, 24]. This dataset was first provided by Xiao et al., and the detailed dataset collection process is available in iPSW(2L)-PseKNC. In summary, each promoter sequence was obtained by truncating an 81-bp fragment (from − 60 to + 20 relative to the TTS located at 0) in the region near the TSS on the K-12 genome, and the non-promoter sequence part here includes introns, exons and intergenic sequences. Non-promoter sequences were obtained by randomly extracting equally long sequence segments from the non-promoter sequence part of the K-12 genome. The obtained promoter and non-promoter sequences were both processed using the CD-HIT [28] software to ensure that the sequence similarity did not exceed 85%. Finally, the benchmark dataset contains 3382 promoter samples and 3382 non-promoter samples.

Furthermore, following the conventions of previous studies [12, 13, 18, 29], we constructed a novel independent test dataset based on the latest version of promoter data provided by RegulonDB (version 11.1) to validate the generalization performance of our model. To ensure that there is no overlap between the independent test dataset and the benchmark dataset, we first obtained the latest version of promoter data from RegulonDB and then removed the promoter samples that appeared in the benchmark dataset. Specifically, we compared the promoter sequences in the benchmark dataset with those in the independent test dataset through a two-layer traversal process. For each sequence, we conducted a pairwise comparison. If an exact match was found between a sequence in the test dataset and any sequence in the benchmark dataset, it indicated that the sample was already present in the benchmark dataset. Consequently, we removed such duplicated promoter sequence samples from the independent test dataset. Finally, we applied CD-HIT software with a same threshold of 0.85 to remove redundant data from the remaining samples. The independent test dataset consists of a total of 395 promoter samples.

In *E. coli*, the promoter is recognized by six σ factors with different regulatory effects. According to this, the types of promoters can be divided into six categories, which are ${\sigma }^{24}, {\sigma }^{28}, {\sigma }^{32}, {\sigma }^{38}, {\sigma }^{54} \text{ and }{ \sigma }^{70}$ respectively. Additionally, depending on the strength of transcription activation and expression, promoters can be classified as strong or weak promoter strengths. Therefore, we provide a comprehensive description of the distribution of promoter samples in both datasets in terms of promoter types and promoter detailed information about the benchmark dataset and the independent test dataset is presented in Table 1, and the total number of samples in the category corresponding to the promoter is also recorded together in the table.

**Table 1:** Details of the *E. coli* promoter dataset

Dataset	Activity	Promoter							Non promoter	All
		${\sigma }^{24}$	${\sigma }^{28}$	${\sigma }^{32}$	${\sigma }^{38}$	${\sigma }^{54}$	${\sigma }^{70}$	${\sigma }^{unknown}$		
Benchmark dataset	Strong	68	10	61	116	17	758	561	/	1591
	Weak	418	123	222	41	74	886	27	/	1791
		486	133	283	157	91	1644	588	/	
		3382							3382	
Independent test dataset	Strong	4	0	12	74	5	69	47	/	211
	Weak	30	5	17	11	0	98	8	/	169
	Confirmed	1	0	0	9	0	1	4	/	15
		35	5	29	94	5	168	59	/	
		395							0	

### Longformer embedding

With the continuous development of the NLP field, especially the large language model (LLM) with the theme of attention mechanism [30], many researchers in the field of bioinformatics have begun to transplant the concept of NLP to bioinformatics problems, such as DNABERT [31]. Longformer, a variant of the Transformer architecture, has been specifically designed to handle long sequential data and has also found application in sequence-to-sequence tasks. Longformer’s attention mechanism is a combination of windowed local-context self-attention and task-inspired global attention. This unique combination enables the Longformer to retain global contextual information while also introducing a focus on deep features within sequences. To mitigate computational costs and complexity, Longformer employs a sparse attention mechanism. This implies that each position calculates attention with only a small subset of other positions, rather than the entire sequence. Longformer has demonstrated promising performance in certain bioinformatics tasks [32, 33]. Inspired by this, we incorporate Longformer into our research question. The results indicate that the utilization of the Longformer enhances predictive performance.

In our study, we use a pre-trained Longformer model to obtain embeddings of promoter DNA sequences. Specifically, we utilize the pre-trained model named “longformer-base-4096”, which supports text sequences up to a maximum length of 4096 and can embed each word into a vector of 768 dimensions. The pre-trained model can be downloaded from Hugging Face [34], and the specific download link is https://huggingface.co/allenai/longformer-base-4096/tree/main.

Although Longformer is developed for long text content processing, the experimental results show that the pre-trained model is still highly effective for our research problem. Perhaps this is because the pre-trained Longformer model captures additional long-term dependence information with a larger field of view, which is particularly helpful for identifying promoter sequences. A point worth stating is that when using BERT-like pre-trained models, researchers usually add special tokens to the input text sequence, such as CLS tokens placed at the beginning of the text to represent the sentence vector. We assume that these special tokens do not have real biological significance and therefore do not add them to the DNA sequence.

### Feature extraction

In NLP, CNN and LSTM have been widely used by researchers to extract text features. Due to this characteristic, CNN and LSTM have also been used in bioinformatics to extract feature vectors from genomic sequences. In our study, we use a three-layer one-dimensional CNN for extracting local features of promoter DNA sequences and a BiLSTM to obtain global features.

Table 2 summarizes the detailed parameters of all components in the three one-dimensional CNN layers in the order of module connection. For each CNN layer, we use the structural order of CBAPD to build the network (C, B, A, P, and D refer to the convolutional layer, batch normalization layer, activation function, pooling layer, and dropout layer, respectively). Here, the ReLU function is used as the activation function of CNN. To reduce tensor dimensions and prevent overfitting, we connect a max pooling layer. The kernel size and stride of each max pooling layer are set to 3 and 1, respectively. To avoid overfitting, we also add dropout layers for the first two convolutional layers, with a random dropout probability of 0.8. For the parameters of the three convolutional layers, the number of convolutional kernels is set to 128, 64, and 32, and their kernel sizes are set to 8, 8, and 3, respectively, with a stride of 2 for each layer.

**Table 2:** Hyperparameters of all components in each convolutional layer

CNN layer	Conv1d	BatchNorm1d	MaxPool1d	Dropout
Layer-1	Filter = 128 Kernel size = 8 Stride = 2	Num Features = 128	Kernel size = 3 Stride = 1	*p* = 0.8
Layer-2	Filter = 64 Kernel size = 8 Stride = 2	Num Features = 64	Kernel size = 3 Stride = 1	*p* = 0.8
Layer-3	Filter = 32 Kernel size = 3 Stride = 2	Num Features = 32	Kernel size = 3 Stride = 1	Not used

### Model setting and evaluation metrics

In this study, we used Python 3, PyTorch framework, and Hugging Face toolkit transformers 4.11.3 for the implementation of iProL. The source code for the implementation of the model for identifying promoters is available at https://github.com/20032303092/iProL. We conducted the model training on the Ubuntu system and utilized CUDA to accelerate the training process. The model was trained for a total of 250 epochs with a batch size of 32, and the initial learning rate was set to 0.0005. The cross-entropy loss function was used to calculate the model error, and the Adam optimization algorithm was used to update the model weights. Additionally, we used the PyTorch StepLR strategy to adjust the learning rate to achieve the optimal model. During the training process, the learning rate decayed by 0.6 times every 50 epochs.

To objectively and fairly evaluate the performance of our proposed promoter predictor iProL, we adopted the five-fold cross-validation and five widely accepted evaluation metrics based on Chou's five-step rule [35]. The five evaluation metrics are sensitivity (Sn), specificity (Sp), accuracy (Acc), Matthews correlation coefficient (MCC), and area under the receiver operating characteristic curve (AUC) [36, 37]. Sn and Sp represent the prediction ability of the predictor for positive and negative samples, respectively. Acc measures the prediction accuracy of the predictor. MCC describes the correlation coefficient between the true classification and predicted classification. The receiver operating characteristic curve reflects the relationship between Sn and Sp at different thresholds, and the AUC value closer to 1 indicates better model performance. The formulas for calculating Sn, Sp, Acc, and MCC are shown below:

1 $$Sn = \frac{TP}{{TP + FN}},$$

2 $$Sp = \frac{TN}{{TN + FP}},$$

3 $$Acc = \frac{TP + TN}{{TP + FN + TN + FP}},$$

4 $$MCC = \frac{TP \times TN - FP \times FN}{{\sqrt {(TP + FP) \times (TP + FN) \times (TN + FN) \times (FN + FP)} }},$$

where TP, TN, FP, and FN represent true positive (correctly predicted positive sample number), true negative (correctly predicted negative sample number), false positive (incorrectly predicted positive sample number), and false negative (incorrectly predicted negative sample number), respectively.

### Sequence analysis and model interpretation

Understanding how the model works is of significant importance for validating its reliability. With the widespread application of interpretability in deep learning methods, we have developed a great interest in the logic behind iProL’s accurate predictions. Therefore, in our research, we employed motif analysis tools such as WebLogo [38] and STREME [39], along with the interpretability method called LIME [40], to analyze our model. By utilizing WebLogo and STREME, we were able to identify enriched motif patterns within DNA sequences. LIME allowed us to generate explanations for specific DNA sequence samples, thereby enabling us to analyze the interpretability of the model's predictions for those samples. Specifically, for the input promoter DNA sequences, we focused on the 2-mer fragments that the model emphasized during the prediction process. We compared these fragments with the identified enriched motif patterns to explore the extent of their alignment, aiming to enhance our understanding of the model and validate its reliability.

## Results and discussion

### Sequence analysis

**Fig. 2:**
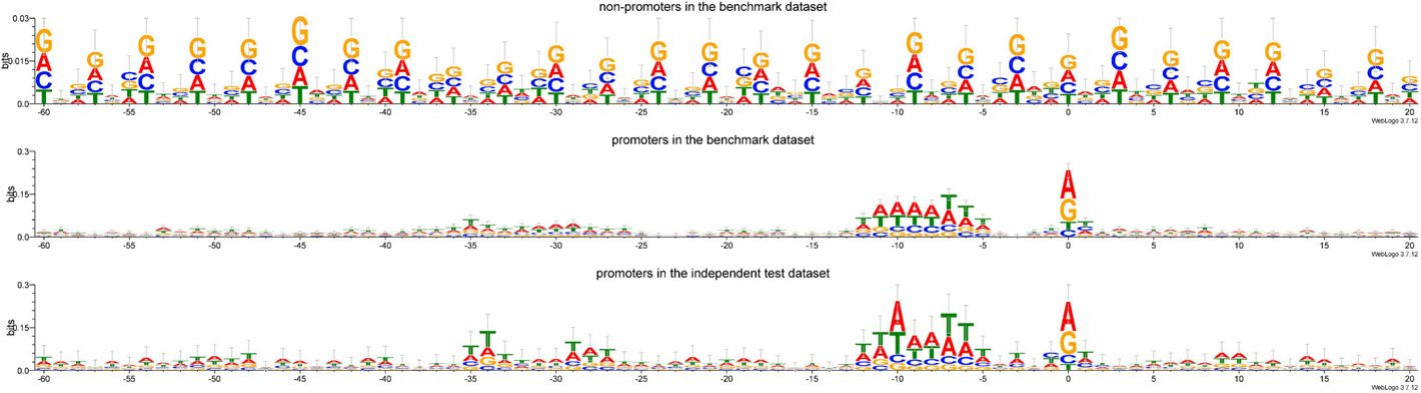
Analysis of sequences using WebLogo

Understanding and analyzing the consensus motif of DNA sequences has positive significance for the identification of promoter sequences. To analyze the nucleotide distribution of promoter sequences, we generated the corresponding sequence logos for the non-promoter sample set and promoter sample set in the benchmark dataset, and all samples in the independent test dataset, respectively. Figure 2 reveals the conserved regions of promoter DNA sequences, where the x-axis subscript 0 represents the TSS, and the y-axis represents the conservation score. As shown in Fig. 2, the consensus motifs of promoter sequences are mainly enriched around the − 10 to − 5 region and the − 35 to − 30 region, as well as the TSS. On the other hand, the conservation score of non-promoter sequences is significantly lower than those of promoter samples, and there are no representative conserved regions present.

### The effect of different embedding methods

Since our promoter input sequence is only 81bp long, it is not comparable to long text sequences. In addition, Longformer is specifically designed for long text processing, while BERT handles text sequences with a maximum length of 512. Therefore, we discuss the classification effect of both pre-trained models, Longformer and BERT. Therefore, we conducted a comparative study of the classification performance of Longformer and BERT, using the most popular BERT pre-training model, bert-base-multilingual-cased, available on the Hugging Face platform. The size of Kmer also had an impact on the experimental results. We found that the BERT pre-trained model's vocabulary only fully maps individual nucleotides, while the Longformer pre-trained model also supports 2-mer mapping. Therefore, we designed three experiments in total, and Table 3 lists all the experimental results. When using 1-mer, Longformer outperformed BERT in terms of Acc, MCC, and AUC scores by 0.73%, 1.09%, and 0.43%, respectively. This indicates that the Longformer pre-trained model using the new form of attention mechanism has an advantage over BERT for the promoter sequence recognition problem.

**Table 3:** Comparison of two pre-trained models using Longformer and BERT

Model	Sn(%)	Sp(%)	Acc(%)	MCC	AUC
BERT, 1-mer	79.51	**90.27**	84.89	0.7021	0.9164
Longformer, 1-mer	83.97	87.26	**85.62**	**0.7130**	0.9207
Longformer, 2-mer (iProL)	**84.62**	86.61	**85.62**	**0.7130**	**0.9211**

Bold values indicate the highest score in the column

When using 2-mer, the Sn and Sp scores are further drawn together, which indicates that the model has a more balanced ability to identify positive and negative samples. In summary, using 2-mer to divide the sequences and using the Longformer pre-trained model as the embedding layer helps us to obtain a better and more balanced promoter recognition tool.

### Discussion on the effects of each module of the model

To ensure that an optimal model can be obtained, we also discussed the impact of other module parts in the iProL model,including the CNN layer and BiLSTM layer. In more detail, inspired by the model design of SPEID [41], we also added a comparative experiment on the location of the batch normalization layer to compare the influence of CBAPD and CAPBD network structure order on the experimental results. Tables 4, 5 and 6, respectively, record the performance of different combinations of three different modules, as analyzed below.

**Table 4:** Effect of different numbers of CNN layers on the performance of the model

Model	Sn(%)	Sp(%)	Acc(%)	MCC	AUC
CNN ×0	82.55	**87.14**	84.85	0.6981	0.9140
CNN ×1	84.12	86.72	85.42	0.7095	0.9196
CNN ×2	84.00	86.40	85.20	0.7048	0.9198
CNN ×3 (iProL)	**84.62**	86.61	**85.62**	**0.7130**	**0.9211**

Bold values indicate the highest score in the column

**Table 5:** Effect of the BiLSTM module on the performance of the model

Model	Sn(%)	Sp(%)	Acc(%)	MCC	AUC
BiLSTM ×0	82.55	**87.14**	84.85	0.6981	0.9140
BiLSTM ×1 (iProL)	**84.62**	86.61	**85.62**	**0.7130**	**0.9211**

Bold values indicate the highest score in the column

**Table 6:** Effect of batch normalization layer location on the performance of the model

Model	Sn(%)	Sp(%)	Acc(%)	MCC	AUC
CAPBD	82.52	**86.66**	84.59	0.6926	0.9125
CBAPD (iProL)	**84.62**	86.61	**85.62**	**0.7130**	**0.9211**

Bold values indicate the highest score in the column

First, to explore the influence of the number of CNN layers on the prediction results, we construct three comparative models except for iProL. These models contain 0, 1, and 2 one-dimensional CNN layers, respectively, and the scores of the four models on all metrics are shown in Table 4. It can be seen that although the performance of the four models is close to each other, iProL constructed by three-layer one-dimensional CNN wins the biggest advantage. Second, for the role played by the BiLSTM layer, we designed a comparison model without the BiLSTM layer. The experimental results are shown in Table 5. Except for Sp, iProL is higher than the comparison model in Sn, Acc, MCC, and AUC by 0.93%, 0.77%, 1.49%, and 0.71%, respectively. We believe that BiLSTM effectively extracts the long-term dependence information of the promoter sequences, which brings better prediction performance to the model. Finally, Table 6 records the experimental results of CAPBD and CBAPD, and the experiment shows that the CBAPD sequential structure is more conducive to the model's recognition of the promoter sequence. To sum up, iProL, composed of different optimal modules, can bring us the best prediction performance.

### Performance comparison on the benchmark dataset

To ensure an objective and unbiased evaluation of our proposed prediction model, on the premise of ensuring that the benchmark dataset is consistent, we compared the model with the method proposed by Xiao et al. and three recently published methods. The four methods are iPSW(2L)-PseKNC, iPromoter-ET, dPromoter-XGBoost and BERT-Promoter. The final comparison is shown in Table 7, where the symbol “/” indicates the missing value. It is worth noting that the method BERT-Promoter used ten-fold cross-validation to compare with the previous methods, so we adopted the experimental results of BERT-Promoter in the comparison.

**Table 7:** Performance comparison of predictors using fivefold cross-validation on benchmark dataset

Predictor	Sn(%)	Sp(%)	Acc(%)	MCC	AUC
iPSW(2L)-PseKNC	81.37	84.89	83.13	0.6630	0.9054
iPromoter-ET	84.23	86.04	85.14	0.7030	0.9193
dPromoter-XGBoost	**85.72**	81.92	83.81	0.6770	/
BERT-Promoter	84.34	86.56	85.45	/	0.9020
iProL (ours)	84.62	**86.61**	**85.62**	**0.7130**	**0.9211**

Bold values indicate the highest score in the column

First of all, in terms of scores, our method achieved the highest scores on Sp, Acc, MCC, and AUC, with 86.61%, 85.62%, 0.7130, and 0.9211, respectively. From the perspective of Sn and Sp, iPSW(2L)-PseKNC focuses on the recognition of positive samples, dPromoter-XGBoost focuses on the recognition of negative samples, while our predictor has more balanced recognition performance and good recognition ability for both positive and negative samples. Secondly, compared with the latest two classifiers, dPromoter-XGBoost and BERT-Promoter, iProL outperforms these two predictors in all aspects except that it is weaker than dPromoter-XGBoost in Sn. Specifically, iProL is 1.91% higher than BERT-Promoter on AUC, 4.69%, and 3.6% higher than dPromoter-XGBoost on Sp and MCC, respectively. In addition, compared to the remaining two predictors, iProL completely outperforms iPromoter-ET and iPSW(2L)-PseKNC, scoring a maximum of 5% higher. To sum up, our iProL is better than the previous methods in most metrics and has a more balanced prediction performance. This suggests that our proposed iProL has a positive significance in predicting promoters.

### Performance comparison on the independent test dataset

To further demonstrate the generalization capability of our model, we conducted independent testing on the independent test dataset and compared it with iPromoter-2L, iPromoter-BnCNN and PredPromoter-MF(2L). Comparisons with other existing methods were not made here because the source code they provide is not successfully run or the web server they provide is no longer available, while iPromoter-2L, iPromoter-BnCNN and PredPromoter-MF(2L) are the three available methods we found whose source code provides a runnable python script. The experimental results on the independent test dataset are shown in Table 8. Since the independent test dataset only contains positive samples, the results are recorded in the format of "TP/FP" in each cell, where TP represents the number of correctly identified promoters and FP represents the number of incorrectly identified promoters. From the experimental results, it can be concluded that the recognition rates of promoters by iPromoter-2L, iPromoter-BnCNN, PredPromoter-MF(2L) and iProL predictors on the independent test dataset are 87.59%, 91.14%, 91.39% and 92.91%, respectively. Our proposed iProL method achieves the highest recognition accuracy among the four predictors.

**Table 8:** Performance comparison between iPromoter-2L, iPromoter-BnCNN, PredPromoter-MF(2L) and iProL on the independent test dataset (TP/FP)

Predictor	${\sigma }^{24}$	${\sigma }^{28}$	${\sigma }^{32}$	${\sigma }^{38}$	${\sigma }^{54}$	${\sigma }^{70}$	${\sigma }^{unknown}$	All
iPromoter-2L	25/10	5/0	27/2	93/1	2/3	152/16	42/17	346/49
iPromoter-BnCNN	30/5	5/0	26/3	91/3	2/3	156/12	50/9	360/35
PredPromoter-MF(2L)	31/4	5/0	26/3	86/8	3/2	159/9	50/8	361/34
iProL (ours)	30/5	5/0	26/3	91/3	4/1	159/9	52/7	**367/28**

Bold value indicates the best performance

### Interpretation

So far, the results have demonstrated the excellent performance of our model in identifying promoter sequences. To gain insights into the driving features behind the model predictions, we employed the model interpretability technique LIME to identify key 2-mer fragments that are important for prediction. The 2-mer fragment is chosen because our model input is a 2-mer mapping. In summary, LIME helps us to deeply understand the behavior and decision-making process of iProL, while providing intuitive visual interpretation of the prediction results.

**Fig. 3:**
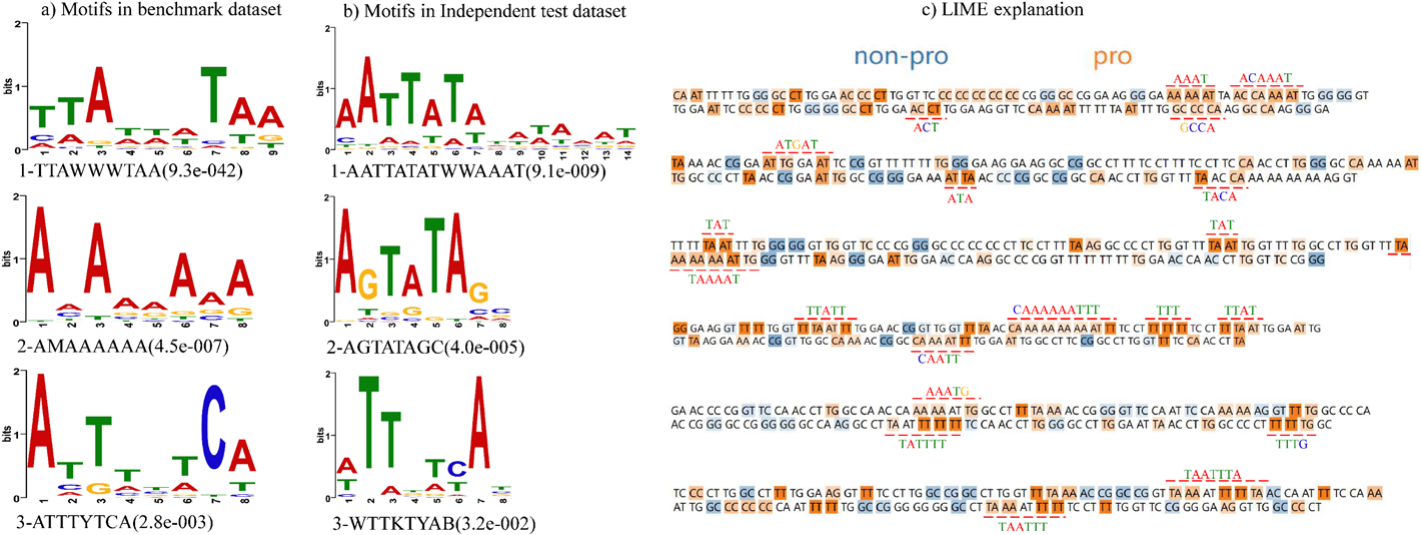
Motifs detected by STREME and LIME visualization explanation. It includes the consensus motifs detected by STREME in the benchmark dataset and the independent test dataset (**a**, **b**), and the visual explanations obtained using LIME on six promoter sequence samples (**c**)

Initially, to further explore the relatively enriched motif patterns in promoter sequences compared to non-promoter sequences, we set the non-promoter sample set as the control sequences and utilized the motif discovery tool STREME to accurately estimate the statistical significance of motifs using Fisher exact test. Figure 3a, b present the top three important consensus motifs discovered by STREME in the benchmark dataset and the independent test dataset, respectively. The sequence composition and *p* value of the corresponding motifs are shown below each motif logo. Supplementary Table S1 provides detailed explanations for non-DNA base letters, while the complete set of consensus motifs in the benchmark dataset and the independent test dataset can be found in Supplementary Figs. S1 and S2. Next, we selected the promoter samples in the independent test set where only iProL identified correctly for model interpretation and used LIME to compute feature weights for the 2-mer fragments at each position to determine the fraction with the greatest impact on the final results. Figure 3c illustrates the preference of our prediction model for 2-mer fragments, and an analytical comparison reveals that promoter sequences are highly enriched in motifs containing base A and base T. Additionally, the sequence patterns (reconstructed from consecutive 2-mer fragments and indicated by red lines in the Fig. 3c) of interest to our model closely align with the consensus motifs revealed by STREME. This indicates that our model focuses on the consensus motifs that hold crucial significance for predictions and produces reliable and accurate predictions.

## Conclusion

In this study, we propose a novel prediction tool, iProL, which first utilizes the Longformer pre-trained model with attention mechanism as the embedding layer, then uses CNN and BiLSTM to extract sequence local features and long-term dependency information, and finally obtains the prediction results through two fully connected layers, achieving state-of-the-art performance. In particular, the successful application of the pre-trained Longformer model in the promoter recognition problem further confirms the availability of BERT-like pre-trained models learned from human natural language data in the field of bioinformatics, indicating that there may be some consensus between genomic language and human language. In addition, through a series of analyses, we find that each module of iProL has a positive effect on the promoter recognition task. Compared with the current state-of-the-art methods, our method has better prediction performance, which provides the possibility for detecting new promoters. To further extend the applicability of our model, we will focus on optimizing the model framework in the future, hoping to successfully apply iProL to related problems such as promoter type identification and strength identification.

### Supplementary Information

Supplementary material 1

## Data Availability

The datasets supporting the conclusions of this article are included with article. Project name: iProL. Project home page: https://github.com/20032303092/iProL. Project inclusion: All datasets and the code needed to replicate the experiment.

## References

[1] Haberle V, Lenhard B (2016). Promoter architectures and developmental gene regulation. Semin Cell Dev Biol.

[2] Browning DF, Busby SJ (2016). Local and global regulation of transcription initiation in bacteria. Nat Rev Microbiol.

[3] Yella VR, Kumar A, Bansal M (2018). Identification of putative promoters in 48 eukaryotic genomes on the basis of DNA free energy. Sci Rep.

[4] Feklístov A, Sharon BD, Darst SA, Gross CA (2014). Bacterial sigma factors: a historical, structural, and genomic perspective. Annu Rev Microbiol.

[5] Ramprakash J, Schwarz FP (2008). Energetic contributions to the initiation of transcription in *E. coli*. Biophys Chem.

[6] Zhang M, Jia C, Li F, Li C, Zhu Y, Akutsu T, Webb GI, Zou Q, Coin LJM, Song J (2022). Critical assessment of computational tools for prokaryotic and eukaryotic promoter prediction. Brief Bioinform.

[7] Wang S, Cheng X, Li Y, Wu M, Zhao Y (2018). Image-based promoter prediction: a promoter prediction method based on evolutionarily generated patterns. Sci Rep.

[8] Lin H, Liang ZY, Tang H, Chen W (2019). Identifying Sigma70 promoters with novel pseudo nucleotide composition. IEEE/ACM Trans Comput Biol Bioinform.

[9] He W, Jia C, Duan Y, Zou Q (2018). 70ProPred: a predictor for discovering sigma70 promoters based on combining multiple features. BMC Syst Biol.

[10] Patiyal S, Singh N, Ali MZ, Pundir DS, Raghava GP (2022). Sigma70Pred: a highly accurate method for predicting sigma70 promoter in Escherichia coli K-12 strains. Front Microbiol.

[11] Liu B, Yang F, Huang DS, Chou KC (2018). iPromoter-2L: a two-layer predictor for identifying promoters and their types by multi-window-based PseKNC. Bioinformatics.

[12] Zhang M, Li F, Marquez-Lago TT, Leier A, Fan C, Kwoh CK, Chou KC, Song J, Jia C (2019). MULTiPly: a novel multi-layer predictor for discovering general and specific types of promoters. Bioinformatics.

[13] Amin R, Rahman CR, Ahmed S, Sifat MHR, Liton MNK, Rahman MM, Khan MZH, Shatabda S (2020). iPromoter-BnCNN: a novel branched CNN-based predictor for identifying and classifying sigma promoters. Bioinformatics.

[14] Lyu Y, He W, Li S, Zou Q, Guo F (2021). iPro2L-PSTKNC: a two-layer predictor for discovering various types of promoters by position specific of nucleotide composition. IEEE J Biomed Health Inform.

[15] Shujaat M, Wahab A, Tayara H, Chong KT (2020). pcPromoter-CNN: a CNN-based prediction and classification of promoters. Genes (Basel).

[16] Bernardino M, Beiko R. Genome-scale prediction of bacterial promoters. In: 2021 IEEE conference on computational intelligence in bioinformatics and computational biology (CIBCB). 2021. 01–08.

[17] Bhukya R, Kumari A, Amilpur S, Dasari CM (2022). PPred-PCKSM: a multi-layer predictor for identifying promoter and its variants using position based features. Comput Biol Chem.

[18] Wang M, Li F, Wu H, Liu Q, Li S (2022). PredPromoter-MF(2L): a novel approach of promoter prediction based on multi-source feature fusion and deep forest. Interdiscip Sci.

[19] Xiao X, Xu ZC, Qiu WR, Wang P, Ge HT, Chou KC (2019). iPSW(2L)-PseKNC: a two-layer predictor for identifying promoters and their strength by hybrid features via pseudo K-tuple nucleotide composition. Genomics.

[20] Le NQK, Yapp EKY, Nagasundaram N, Yeh HY (2019). Classifying promoters by interpreting the hidden information of DNA sequences via deep learning and combination of continuous FastText N-Grams. Front Bioeng Biotechnol.

[21] Tayara H, Tahir M, Chong KT (2020). Identification of prokaryotic promoters and their strength by integrating heterogeneous features. Genomics.

[22] Liang Y, Zhang S, Qiao H, Yao Y (2021). iPromoter-ET: identifying promoters and their strength by extremely randomized trees-based feature selection. Anal Biochem.

[23] Li H, Shi L, Gao W, Zhang Z, Zhang L, Zhao Y, Wang G (2022). dPromoter-XGBoost: detecting promoters and strength by combining multiple descriptors and feature selection using XGBoost. Methods.

[24] Le NQK, Ho QT, Nguyen VN, Chang JS (2022). BERT-Promoter: an improved sequence-based predictor of DNA promoter using BERT pre-trained model and SHAP feature selection. Comput Biol Chem.

[25] Devlin J, Chang M-W, Lee K, Toutanova K. Bert: pre-training of deep bidirectional transformers for language understanding. 2018. arXiv:181004805.

[26] Beltagy I, Peters ME, Cohan A. Longformer: the long-document transformer. 2020. arXiv:200405150.

[27] Gama-Castro S, Salgado H, Santos-Zavaleta A, Ledezma-Tejeida D, Muñiz-Rascado L, García-Sotelo JS, Alquicira-Hernández K, Martínez-Flores I, Pannier L, Castro-Mondragón JA (2016). RegulonDB version 9.0: high-level integration of gene regulation, coexpression, motif clustering and beyond. Nucl Acids Res.

[28] Fu L, Niu B, Zhu Z, Wu S, Li W (2012). CD-HIT: accelerated for clustering the next-generation sequencing data. Bioinformatics.

[29] Li F, Chen J, Ge Z, Wen Y, Yue Y, Hayashida M, Baggag A, Bensmail H, Song J (2021). Computational prediction and interpretation of both general and specific types of promoters in *Escherichia coli* by exploiting a stacked ensemble-learning framework. Brief Bioinform.

[30] Vaswani A, Shazeer N, Parmar N, Uszkoreit J, Jones L, Gomez AN, Kaiser Ł, Polosukhin I. Attention is all you need. In: Advances in neural information processing systems, vol. 30. 2017.

[31] Ji Y, Zhou Z, Liu H, Davuluri RV (2021). DNABERT: pre-trained bidirectional encoder representations from transformers model for DNA-language in genome. Bioinformatics.

[32] Wang Z, Zhang Y, Yu Y, Zhang J, Liu Y, Zou Q (2023). A unified deep learning framework for single-cell ATAC-seq analysis based on ProdDep transformer encoder. Int J Mol Sci.

[33] Li Y, Wehbe RM, Ahmad FS, Wang H, Luo Y (2023). A comparative study of pretrained language models for long clinical text. J Am Med Inform Assn.

[34] Jain SM. Hugging face. In: Introduction to transformers for NLP: with the hugging face library and models to solve problems. Berlin: Springer; 2022. pp. 51–67.

[35] Chou K-C (2011). Some remarks on protein attribute prediction and pseudo amino acid composition. J Theor Biol.

[36] Fawcett T (2006). An introduction to ROC analysis. Pattern Recogn Lett.

[37] Hanley JA, McNeil BJ (1982). The meaning and use of the area under a receiver operating characteristic (ROC) curve. Radiology.

[38] Crooks GE, Hon G, Chandonia J-M, Brenner SE (2004). WebLogo: a sequence logo generator. Genome Res.

[39] Bailey TL (2021). STREME: accurate and versatile sequence motif discovery. Bioinformatics.

[40] Ribeiro MT, Singh S, Guestrin C. "Why should I trust you?" Explaining the predictions of any classifier. In: Proceedings of the 22nd ACM SIGKDD international conference on knowledge discovery and data mining. 2016. pp. 1135–44.

[41] Singh S, Yang Y, Póczos B, Ma J (2019). Predicting enhancer-promoter interaction from genomic sequence with deep neural networks. Quant Biol.

