# Supplementary material for: iProL: identifying DNA promoters from sequence information based on Longformer pre-trained model
Source: BMC Bioinformatics. 2024 Jun 25;25:224. doi: 10.1186/s12859-024-05849-9 (PMC11201334; doi:10.1186/s12859-024-05849-9)
Supplement: Supplementary file 1 — Supplementary material 1 [file 12859_2024_5849_MOESM1_ESM.docx]

iProL: identifying DNA promoters from sequence information based on Longformer pre-trained model

Binchao Peng^1^[, Guicong Sun](#_bookmark5)^1^[, Yongxian Fan^1,*^](#_bookmark2)

^1^ School of Computer Science and Information Security, Guilin University of Electronic Technology, Guilin 541004, China

**Supplementary Table S1** DNA Alphabet.

| **Symbol(s)** | **Name** | **Matches** |
| --- | --- | --- |
| V | Not T | A C G |
| B | Not A | C G T |
| M | Amino | A C |
| W | Weak | A T |
| Y | Pyrimidine | C T |
| K | Keto | G T |

Each symbol represents the base it matches, for example, the symbol "V" means it can match one of the three bases A, C and G.


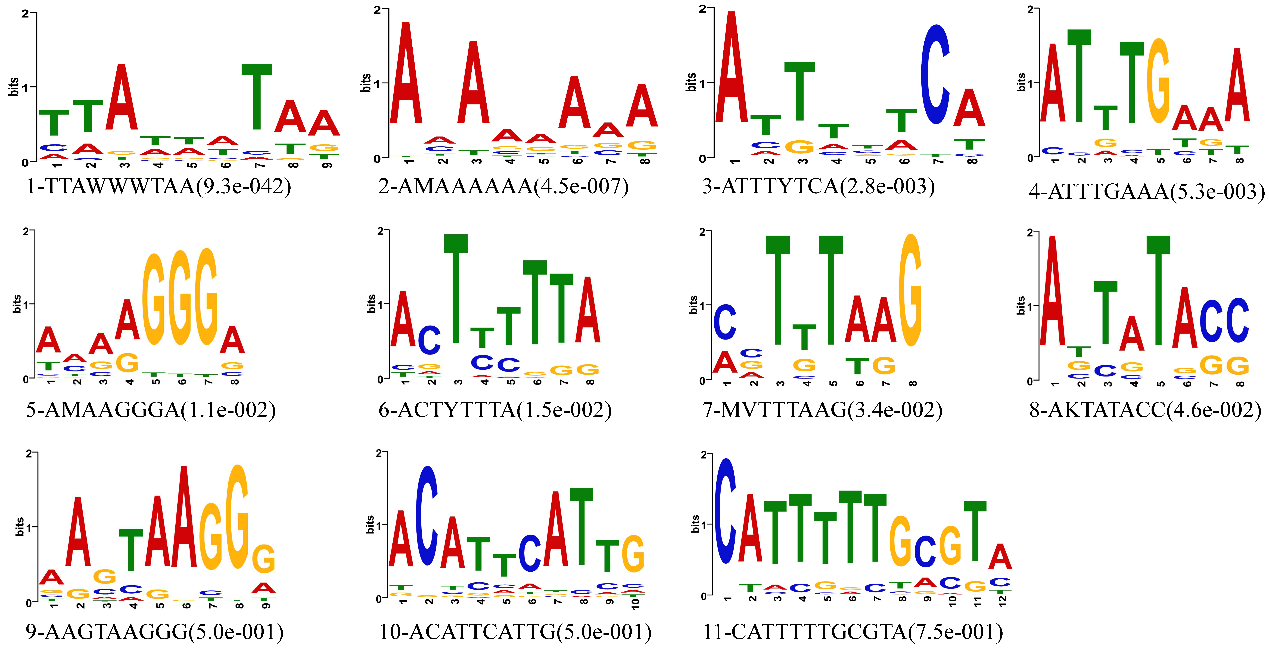


**Supplementary Fig. S1** The complete set of consensus motifs in the benchmark dataset.


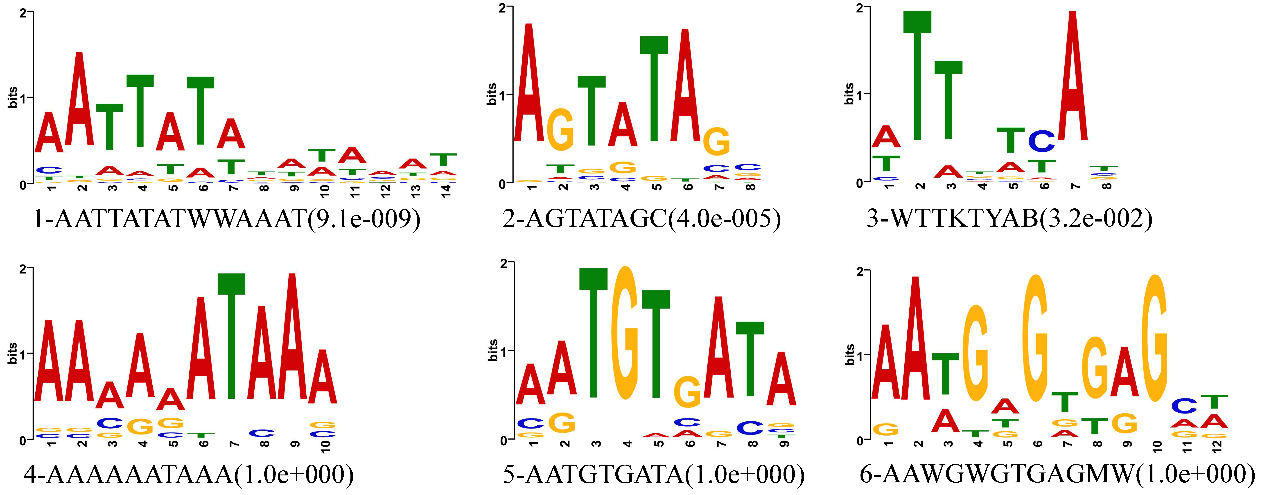
**Supplementary Fig. S2** The complete set of consensus motifs in the independent test dataset.
